# Nanomodified Switch Induced Precise and Moderate Activation of CAR‐T Cells for Solid Tumors

**DOI:** 10.1002/advs.202205044

**Published:** 2023-02-08

**Authors:** Xinyue Wang, Fanyan Meng, Xiang Li, Luxin Xue, Anni Chen, Yuling Qiu, Zhifan Zhang, Lin Li, Fengcen Liu, Yishan Li, Zhichen Sun, Yanhong Chu, Ruihan Xu, Lixia Yu, Jie Shao, Manman Tian, Xiaoping Qian, Qin Liu, Baorui Liu, Rutian Li

**Affiliations:** ^1^ The Comprehensive Cancer Centre of Nanjing Drum Tower Hospital The Affiliated Hospital of Nanjing University Medical School and Clinical Cancer Institute of Nanjing University Nanjing 210008 China; ^2^ Nanjing Drum Tower Hospital Clinical College of Traditional Chinese and Western Medicine Nanjing University of Chinese Medicine Nanjing 210008 China; ^3^ Department of Pathology Nanjing Drum Tower Hospital The Affiliated Hospital of Nanjing University Medical School Nanjing 210008 China

**Keywords:** cancer immunotherapy, cytokine‐release syndrome, nanoparticles, on‐target/off‐tumor effects, chimeric antigen receptor‐T (CAR‐T)

## Abstract

Chimeric antigen receptor (CAR)‐T cell therapy is a transformative treatment against advanced malignancies. Unfortunately, once administrated in vivo, CAR‐T cells become out of artificial control, and fierce response to CAR‐T therapy may cause severe adverse events, represented by cytokine‐release syndrome and on‐target/off‐tumor effects. Here, a nanomodified switch strategy is developed, leading to sustained and precise “on‐tumor only” activation of CAR‐T cells. Here, original gelatinase‐responsive nanoparticles (NPs) are used to selectively deliver the heterodimerizing switch, which is the key component of switchable CAR with separated activation modules. The “NanoSwitch” is tumor‐specific, thus inactivated switchable CAR‐T cells do little harm to normal cells, even if the normal cells express the target of CAR‐T. Owing to the sustained‐release effect of NPs, the CAR‐T cells are activated smoothly, avoiding sudden release of cytokine. These data introduce NanoSwitch as a universal and applicable solution to safety problems of CAR‐T therapy regardless of the target.

## Graphical abstract

Here, nanoparticles based on gelatinase‐responsive strategy are used to construct “NanoSwitch” to control the activation process of chimeric antigen receptor (CAR)‐T cells, leading to on‐site activation of CAR‐T cells at the tumor region in a precise and sustainable way. This platform has promising potential to prevent the main obstacles in the CAR‐T therapy for solid tumors including cytokine‐release syndrome and on‐target/off‐tumor toxicity.

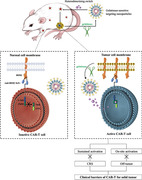

## 1 Introduction

Chimeric antigen receptor (CAR)‐T therapy has dramatically reformed the treatment landscape of hematologic malignancies.^[^ ^1^ ^]^ After genetic engineering, CAR‐T cells can recognize certain tumor ligands and kill the target tumor specifically, thus leading to remarkable therapeutic responses in multiple trials.^[^ ^2^ ^]^ However, once administrated in vivo, traditional CAR‐T cells may become out of artificial control, so rapid and durable clinical responses of CAR‐T therapy could cause severe or even lethal adverse events,^[^ ^3^ ^]^ including two main classifications.^[^ ^4^ ^]^ The first troubling adverse event is toxicities resulting from sudden and bursting release of cytokines, which were reported in nearly all successful clinical trials of CAR‐T cells targeting cluster of differentiation 19 (CD19).^[^ ^2c^ ^]^ Vigorous interactions of CAR‐T cells with tumor cells and immune cells from the host can lead to drastic activation and expansion of CAR‐T cells, which may cause cytokine‐release syndrome (CRS).^[^ ^5^ ^]^ The second is on‐target/off‐tumor effects induced by interactions between the CARs and the target antigens expressed by nonmalignant cells, as most targets of CAR‐T for solid tumors have some degree of shared expression with normal tissues.^[^ ^6^ ^]^ Typically, human epidermal growth factor receptor‐2 (HER2) is one of the most widely used CAR‐T targets in solid tumors.^[^ ^7^ ^]^ CAR‐T cell therapy targeting HER2 caused fatal toxicity in the first patient treated, due to little notice of HER2 expression in the patient's lung epithelial cells, which can be markedly recognized by CAR‐T cells. The patient experienced respiratory distress within 15 min and died 5 days after treatment.^[^ ^8^ ^]^ Recently, a growing number of strategies have been designed to improve security of CAR‐T,^[^ ^9^ ^]^ but consequently make CAR‐T cells extremely difficult to fabricate.^[^ ^4^ ^]^

To control CAR‐T cells in a more effective and simplified way, based on the gelatinase‐sensitive targeting strategy, we developed a nanomodified switch (hereinafter referred to as NanoSwitch), leading to a sustained and precise activation of CAR‐T cells. The key components of switchable CAR‐T (hereinafter referred to as CAR‐T) were separated into two polypeptides,^[^ ^10^ ^]^ which activated only when the heterodimerizing switch (we used rapamycin as switch in this study) is present. Without switch, the CAR‐T cells were not able to be activated either in vitro or in vivo. The sequential delivery of the switch could control the activation of the CAR‐T. However, merely delivery of switch in a systemic manner could not overcome the on‐target/off‐tumor effect as the switch will also distribute into the nontumoral region.

**Figure 1:**
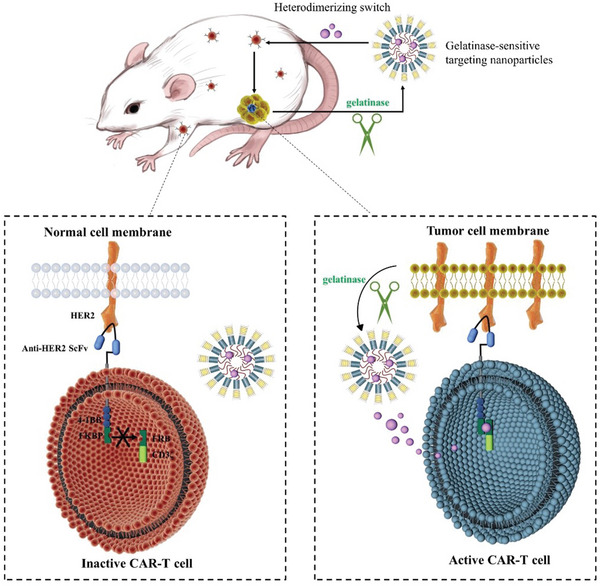
Schematic diagram of tumor‐specific activation of switchable CAR‐T with gelatinase‐responsive NanoSwitch.

In order to resolve this problem, we constructed a nanomodified switch by making use of gelatinase. Gelatinases, also known as matrix metalloproteinase (MMP) 2/9, have been reported to be the most important cancer‐related MMPs, which play a vital role in numerous malignant tumor behaviors, especially metastasis.^[^ ^11^ ^]^ MMPs are specifically and highly expressed in gastric cancer microenvironment but seldom expressed in nontumoral tissue.^[^ ^12^ ^]^ Previously, we inserted the gelatinase‐responsive peptide between the methoxy poly(ethylene glycol)–polycaprolactone (mPEG–PCL) copolymers. As gelatinases were extracellularly secreted into the tumor microenvironment, the PEG–PCL conjugates would be cleaved at tumor sites and most incorporated drugs would distribute to the tumor tissue owing to the deformation of the nanoparticles (NPs). The switch molecule was then loaded into the gelatinases‐responsive NPs. Therefore, even if the normal cells express the CAR‐T target, they would not be harmed by CAR‐T cells as the switch molecules do not exist (**Figure** **1**). Moreover, NPs released switch in a sustainable manner, so the CAR‐T cells were activated smoothly, avoiding CRS caused by sudden release of cytokine and prolonging the effectiveness of CAR‐T cells. Our work provided evidence for tumor‐targeting nanodrug as a novel and pervasive approach to overcome the main shortcomings in CAR‐T cells against solid tumors, thereby inducing their antitumor effect in a secure and controllable way for clinical use.

## 2 Results

### 2.1 Preparation of Switchable CAR‐T Cells and NanoSwitch

CAR plasmid (Figure S1a, Supporting Information) was transfected into the T cells successfully (Figure S1b, Supporting Information) with CAR expression lasting for more than 10 days in vitro (Figure S1c, Supporting Information), suggesting the stability of CAR‐T cells. The initial average diameter of NanoSwitch was 266.3 ± 3.6 nm (average and standard deviation of 3 independently prepared batches) with a zeta potential of −0.87 ± 0.26 mV (average and standard deviation of 3 independently prepared batches) at pH 7.4. The diameter kept stable for more than 28 days (Figure S2a, Supporting Information). The drug loading contents of rapamycin were 25.60 ± 0.56%. The encapsulation efficiency of rapamycin was 53.33 ± 0.61%. According to the transmission electron microscope (TEM) observation, the prepared NPs exhibited a spherical‐like shape with a smooth surface, which collapsed morphologically after incubation with gelatinase for 24 h.^[^ ^13^ ^]^ The result demonstrated that the NPs can be sensitively stimulated by gelatinases, which are highly secreted by gastric cancer cells specifically.^[^ ^12^ ^]^

### 2.2 NanoSwitch Activated Switchable Effect of CAR‐T Cells Smoothly In Vitro

**Figure 2:**
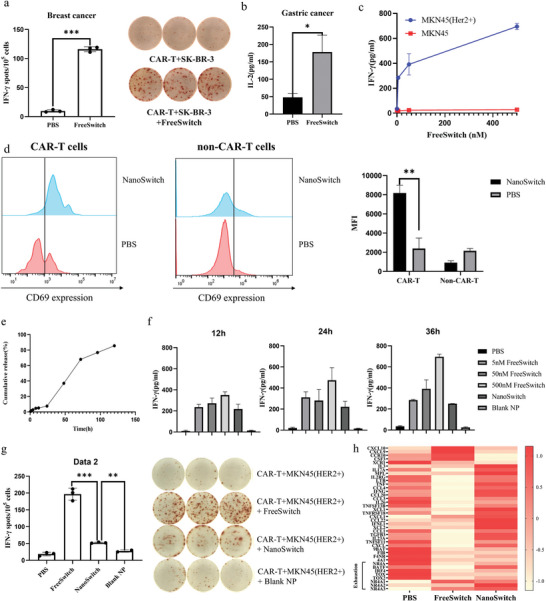
Precise and moderate reaction of NanoSwitch CAR‐T in vitro. a) IFN‐*γ* secretion of switchable CAR‐T cells activated by FreeSwitch after incubation with breast cancer cells SK‐BR‐3, which expressed HER2 naturally. b) IL‐2 secretion of switchable CAR‐T cells activated by FreeSwitch after 24 h incubation with gastric cancer cells MKN45 (HER2+), which expressed HER2 through genetic modification. c) IFN‐*γ* secretion of switchable CAR‐T cells activated by various concentrations of FreeSwitch after incubation with tumor cells MKN45 and MKN45 (HER2+). d) The expression of CD69 on CAR negative and positive subpopulations activated by NanoSwitch after incubation with tumor cells MKN45 (HER2+). e) Cumulative release portion of rapamycin from gelatinase‐responsive nanoparticles over 120 h. Data points represent the mean of at least three experiments. f) IFN‐*γ* secretion of switchable CAR‐T cells activated by different switches after incubation with tumor cells MKN45 (HER2+). The rapamycin concentration of NanoSwitch was 500 nm according to the drug loading content. g) IFN‐*γ* secretion of switchable CAR‐T cells activated by FreeSwitch (500 nm) and NanoSwitch (equivalent to 500 nm) after 24 h incubation with tumor cells MKN45 (HER2+). h) Heat map representing differential expression genes of transfected CAR‐T cells. Immune‐related genes that are different expressed between the FreeSwitch group and NanoSwitch group were partly included (fold change ≥2 and *p* < 0.05), and genes associated with T cell exhaustion are annotated. Data are shown as mean ± standard deviation (SD). Data points represent the mean of at least three experiments. Where the error bars are not readily evident, the SD was lower than the width of the symbols. Student's *t*‐test, ns (not significant), *p* > 0.05, *p < 0.05, **p < 0.01, ***p < 0.001.

CAR‐T cells were cocultured with different forms of switch to treat tumor cells, then we analyzed T lymphocyte activation markers and cytokine secretion to test the switchable effect. Incubated with tumor cells SK‐BR‐3, the CAR‐T cells activated by FreeSwitch secreted more interferon‐gamma (IFN‐*γ*) than the inactivated CAR‐T cells (*p* < 0.001), indicating that the CAR‐T cells can be activated by switch (**Figure** **2**a). To find out whether the switch‐controlled activation was target‐specific, we genetically modified gastric cancer cells MKN45, making them express extracellular and transmembrane fragment of HER2 (Figure S3a, Supporting Information). Interleukin (IL)‐2 (Figure 2b) and IFN‐*γ* (Figure 2c) secretion assay of CAR‐T with MKN45 (HER2+) demonstrated that activation of CAR‐T cells relied on the switch and was in a dose‐dependent manner. On the contrary, the CAR‐T cells incubated with unmodified MKN45, which can be considered as HER2 negative, showed no obvious change in IFN‐*γ* secretion, regardless of the dose of switch molecule (Figure 2c). These results not only proved the target specificity of the switchable effect, but also indicated that the switch activated the reaction between CAR‐T cells and tumor cells, rather than stimulating CAR‐T cells only. CD69 is known as an important T cell activation marker.^[^ ^14^ ^]^ After incubation with NanoSwitch, CD69 was upregulated significantly in CAR positive subpopulation (*p* < 0.01), while remained unchanged in CAR negative subpopulation (Figure 2d), indicating that it was the switchable CAR‐T cells that were specifically activated by NanoSwitch.

Owing to the sustained‐release effect of gelatinase‐responsive NPs, switch was slowly released from NPs in a sustainable manner (Figure 2e). As time goes by, IFN‐*γ* secreted by CAR‐T cells activated by FreeSwitch grew vigorously, while remained stable in the NanoSwitch group (Figure 2f). The enzyme linked immunospot (ELISpot) results also showed that NanoSwitch upregulated cytokine secretion significantly (*p* < 0.001), which was not as fierce as that of FreeSwitch at the same dose (Figure 2g). To further compare the differences of CAR‐T cells activated by different forms of switch and explore the mechanism of its improved therapeutic effect, we performed RNA‐sequencing‐based transcriptome analyses (Figure 2h). Kyoto Encyclopedia of Genes and Genomes (KEGG) enrichment analysis suggested that the differential genes between the FreeSwitch group and the NanoSwitch group were enriched in multiple pathways relating to tumor immunology (Figure S4a, Supporting Information). We found that some T cell exhaustion related genes were differentially expressed between the two groups. Basic leucine zipper ATF‐like transcription factor (BATF) and interferon regulatory factor 4 (IRF4) were critical genes in the inhibition pathway of T cell exhaustion.^[^ ^15^ ^]^ CAR‐T cells activated by NanoSwitch showed increased expression of BATF and IRF4, suggesting that CAR‐T cells activated by NanoSwitch were less exhausted, and thus have a more persistent effect. Above results demonstrated that NanoSwitch activated CAR‐T cells most smoothly and persistently.

### 2.3 Biodistribution and Tumor Targeting of Gelatinase‐Responsive NPs

**Figure 3:**
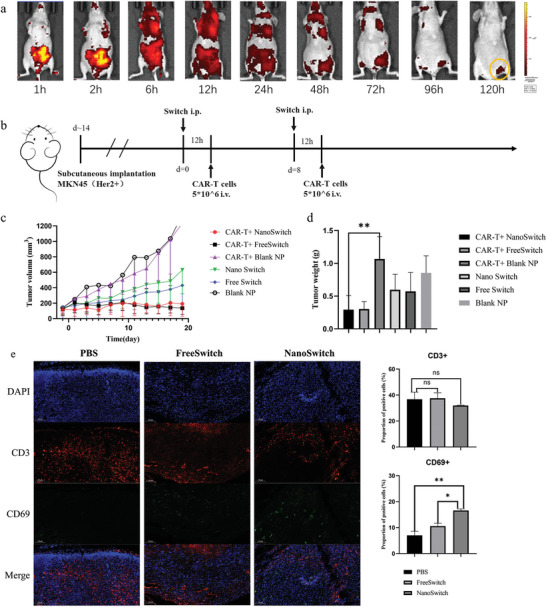
Biodistribution and tumor suppression of NanoSwitch in vivo. a) The dynamics of NIR775 signals over time within tumor tissue after systemically delivering NCBS‐loaded NPs. Tumor site was circled in yellow. b) Schematic illustration of treatment process in subcutaneous tumor model. c,d) Enhanced antitumor effect of NanoSwitch mediated antigen modification cooperative with CAR‐T cell therapy in a subcutaneous MKN45 tumor model. Tumor growth profiles (c) and tumor weight (d) of mice treated with CAR‐T + NanoSwitch, CAR‐T + FreeSwitch, CAR‐T + Blank NP, NanoSwitch, FreeSwitch, Blank NP, respectively. e) Immunofluorescence staining of the tumor samples after the indicated treatment. Anti‐CD3 antibody (red) was used for T cells marker, anti‐CD69 antibody (green) was used for activated T cell marker, and DAPI (4,6‐diamidino‐2‐phenylindole, blue) for nuclear staining of tumor tissues from each group. Scale bar: 100 µm. Data are represented as mean ± SD. Student's *t*‐test, ns (not significant), *p* > 0.05, *p < 0.05, **p < 0.01.

It is crucial to clarify in vivo metabolism and biodistribution of NPs. Bis(trihexylsiloxy)silicon 2,3‐naph‐thalocyanine (NCBS) was carried in the NPs and given to Balb/c nude mice bearing MKN45 subcutaneous tumor. The near‐infrared 775 (NIR775) fluorescent signals were observed on both tumors and abdomen 6 h after intraperitoneal administration. With time going on, the nanoparticles gradually accumulated in tumor because of the deformation and aggregation of the nanoparticles in response to gelatinase in tumor environment. Accordingly, the fluorescence density of other organs decreased, while NIRF signals gradually increased in the tumor. At 96 h after administration, the fluorescence signal could only be observed in the tumor region (**Figure** **3**a). Above results showed the on‐site intelligent targeting of NPs in tumor distribution, thus mediating tumor‐specific activation of switchable CAR‐T.

### 2.4 Switchable Effect of CAR‐T In Vivo

Based on the verification of in vitro activation of switchable CAR‐T and in vivo tumor targeting capabilities of gelatinase‐responsive NPs, we then assessed the in vivo antitumor effect in mice bearing HER2 high‐expressed MKN45 xenograft tumor model (Figure 3b). CAR‐T cells without switch showed little inhibition effect on tumor growth compared with untreated group, suggesting that unswitched CAR‐T cell did no harm to HER2 positive cells (Figure 3c). As expected, switched CAR‐T cells elicited the maximum antitumor efficacy in tumor growth control compared with unswitched CAR‐T. Although FreeSwitch and NanoSwitch alone exhibited some antitumor effect, the difference was not that significant compared with CAR‐T/NanoSwitch and CAR‐T/FreeSwitch groups (Figure 3d), confirming that the antitumor effect was mainly caused by the activated CAR‐T cells. These results demonstrated the switchable effect of the CAR‐T in vivo. Notably, according to Figure 3e, more activated T cells (marked by CD69) were observed inside the tumor region in the NanoSwitch group compared with both the control group and the FreeSwitch group, suggesting that NanoSwitch group inhibited the growth of the tumor by activating T cells more effectively and specifically.

### 2.5 Gelatinase‐Responsive NPs Reduced the Side Effects of Switch of CAR‐T

**Figure 4:**
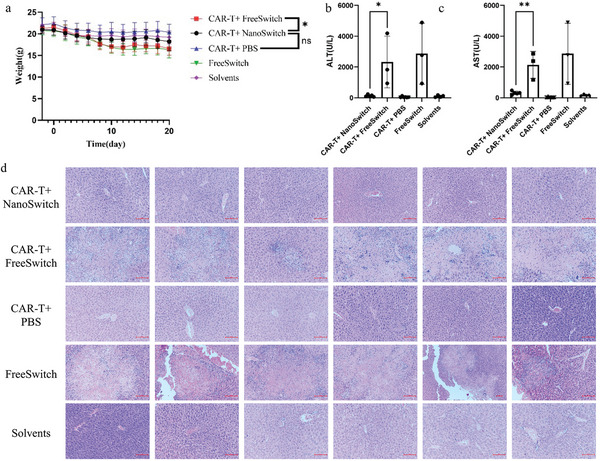
Gelatinase‐responsive NPs reduced the side effects of switch of CAR‐T. a) Weight loss profiles over time after treatment with CAR‐T + NanoSwitch, CAR‐T + FreeSwitch, CAR‐T + phosphate buffered saline (PBS), FreeSwitch, Solvents of FreeSwitch, respectively. b,c) Liver function indexes in 21 days after various treatments. d) Histological analyses of liver toxicity through hematoxylin‐eosin (H&E) staining (×200) after treatment with CAR‐T + NanoSwitch, CAR‐T + FreeSwitch, CAR‐T + PBS, FreeSwitch, Solvents of FreeSwitch, respectively. Scale bar: 100 µm. Data are represented as mean ± SD. Student's *t*‐test, ns (not significant), *p* > 0.05, *p < 0.05, **p < 0.01.

Although FreeSwitch can also activate switchable CAR‐T cells, it caused severe side effects. We found that FreeSwitch decreased the appetite and life vitality of mice, while mice treated with NanoSwitch and CAR‐T/NanoSwitch were as vigorous as control group. Moreover, mice treated with FreeSwitch or CAR‐T/FreeSwitch had lost weight seriously since treatment, while those treated with NanoSwitch or CAR‐T/NanoSwitch maintained vitality and kept their weight at the same level as the control group (**Figure** **4**a). FreeSwitch or CAR‐T/FreeSwitch group showed a remarkable increase in serum concentration of alanine aminotransferase (ALT) and aspartic aminotransferase (AST), suggesting that the FreeSwitch caused liver function damage (Figure 4b,c). As for the liver tissue of CAR‐T/FreeSwitch group, the coagulative necrosis was whitish slight pink in color on gross appearance and elliptical in shape. According to pathological examination, livers of CAR‐T/FreeSwitch group occurred in necrosis and formed the focal cellular necrosis focus (Figure 4d), and lungs of those mice exhibited inflammatory cell infiltration and pulmonary congestion (Figure S5, Supporting Information). Those of CAR‐T/NanoSwitch did not show any pathological injuries (Figure 4d). Blood tests and pathological examination confirmed that gelatinase‐responsive NPs can reduce the side effects of switch of CAR‐T.

### 2.6 CAR‐T Cells Activated by NanoSwitch Released Cytokines More Sustainably In Vivo

**Figure 5:**
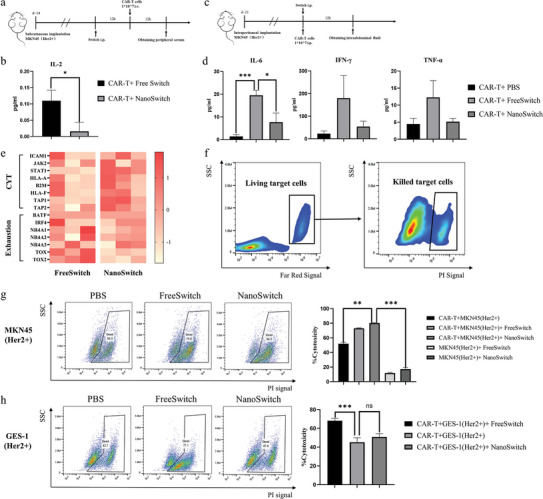
NanoSwitch activated CAR‐T cells more precisely and moderately. a) Schematic illustration of cytokine determination in subcutaneous tumor model. b) Serum cytokine level in subcutaneous tumor model. c) Schematic illustration of cytokine determination in peritoneal metastasis tumor model. d) Levels of cytokine in peritoneal effusion. e) Heat map representing differential expression genes of excised tumor tissue. CYT, genes relating to intratumoral cytolytic activity; Exhaustion, genes relating to T cell exhaustion. Genes involved in tumor antigen presentation (e.g., HLA‐A, HLA‐F) and cytokine signaling in the tumor microenvironment (e.g., JAK2, STAT1) were included. f) The gating methods for flow cytometry analysis of cytotoxicity assays. The living target cells were first stained with cell proliferation kit CellTrace, which is a far red dye with excitation and emission wavelength at 630 and 661 nm, respectively. The dead cells were stained with propidium iodide (PI). g) Evaluating cytotoxicity of tumor cells MKN45 (HER2+) treated with CAR‐T + NanoSwitch, CAR‐T + FreeSwitch, CAR‐T, NanoSwitch, and FreeSwitch. *E*:*T*  =  5:1. h) Evaluating cytotoxicity of gastric mucosal epithelial cells GES‐1 treated with CAR‐T + NanoSwitch, CAR‐T + FreeSwitch, CAR‐T, NanoSwitch, and FreeSwitch. *E*:*T*  =  5:1. Data are shown as mean ± SD. Student's *t*‐test, ns (not significant), *p* > 0.05, *p < 0.05, **p < 0.01, ***p < 0.001.

CRS is the most common adverse event of CAR‐T therapy, because CAR‐T cells usually engage with targets and activate excessively.^[^ ^16^ ^]^ In this study, as a result of the sustained‐release effect of switch by NPs, we found that the levels of serum cytokines in NanoSwitch group were lower than those of FreeSwitch group. In the subcutaneous tumor model, the level of IL‐2 in blood serum from CAR‐T/NanoSwitch group was significantly lower than that of CAR‐T/FreeSwitch group (**Figure** **5**b). In peritoneal metastasis tumor model, several levels of various cytokines in peritoneal effusion were higher in the CAR‐T/FreeSwitch group. IL‐6, which is considered as the key cytokine of CRS,^[^ ^17^ ^]^ increased enormously during 24 h after CAR‐T infusion with FreeSwitch (*p* < 0.001). However, IL‐6 secreted by CAR‐T cells activated by NanoSwitch was significantly less (Figure 5d). These results indicated that our strategy has the potential to prevent overactivation of CAR‐T cells.

Genes relating to intratumoral cytolytic activity (CYT)^[^ ^18^ ^]^ expressed higher in tumor tissue of NanoSwitch group than that of FreeSwitch group (Figure 5e), which reflected the antitumor activity of CAR‐T cells to a certain extent. Compared with FreeSwitch group, NanoSwitch group showed up‐regulated expression of genes that promote antigen presentation, such as transporter (TAP1, TAP2) and major histocompatibility complex class I (HLA‐A, HLA‐F). In addition, the expression of genes related to cytokine signaling such an Janus kinase2 (JAK2) and signal transducer and activator of transcription 1 (STAT1) was also up‐regulated in the TME of NanoSwitch group. This finding coincided with the results of our immunofluorescence staining, suggesting that more actived T cells infiltrated into the tumor tissue of the NanoSwitch group. Moreover, genes promoting T cell exhaustion such as nuclear receptor subfamily 4 group A member (NR4A1, NR4A2, NR4A3) and thymocyte selection associated high mobility group box family member (TOX, TOX2) were downregulated in the NanoSwitch group compared with the FreeSwitch group. Overall, consistent with the other results of our experiments, the RNA sequencing results further proved that NanoSwitch could not only moderate the activation of CAR‐T cells, but also prolong the effect.

### 2.7 NanoSwitch CAR‐T Strategy Is Expected to Be a Solution to Off‐Tumor Effect

On‐target/off‐tumor toxicity is a key problem of CAR‐T therapy when applied to solid tumors.^[^ ^19^ ^]^ Conventional CAR‐T cells circulate throughout the whole body along with blood and can be activated by any cell expressing the CAR‐T target.^[^ ^20^ ^]^ Our NPs have been confirmed to respond to gelatinase specifically and deliver the switch to tumor site selectively,^[^ ^12^ ^]^ minimizing nonspecific stimulation of CAR‐T cells. We prepared human gastric epithelial cell line GES‐1 with transgenic expression of HER2 (Figure S3b, Supporting Information), which simulated normal cells expressing CAR‐T target. FreeSwitch induced CAR‐T cells to kill MKN45 (HER2+) and GES‐1 (HER2+) at similar level. Compared with CAR‐T/PBS and only NanoSwitch groups, CAR‐T/NanoSwitch caused effective cytotoxicity to MKN45 (HER2+), but CAR‐T/NanoSwitch did little harm to GES‐1 (HER2+), which was essentially the same (*p* = 0.1773) as the effect of CAR‐T/PBS (Figure 5g,h). Above results initially proved our hypothesis. CAR‐T cells activated by FreeSwitch were like traditional CAR‐T, which indiscriminately attacked both tumor and normal cells expressing their targets, resulting in on‐target/off‐tumor toxicity. However, owing to the targeted delivery of switch to tumor cells by NPs, switchable CAR‐T cells only killed tumor cells selectively.

## 3 Discussion

The safety issue has always been conspicuous since CAR‐T therapy was applied in the treatment of tumors, especially solid tumors. With the attempt to reform CAR‐T cells, many strategies have been developed to control CAR‐T, such as photodynamic guidance^[^ ^21^ ^]^ or drug inactivation.^[^ ^9b^ ^]^ Unfortunately, most plasmid structures of reformed CAR‐T were much more complicated both economically and temporally, resulting in decreased transfection efficiency and increased production costs.^[^ ^4^ ^]^ Furthermore, most of the reported strategies can only be used under a fixed circumstance. An unsophisticated and “fits‐all” strategy is undoubtedly needed.

In this study, we attempted to solve this problem by means of nanodrug delivery system. We understood the interaction between CAR‐T cells and the immune system as well as nanosystem in vivo, based on which the NanoSwitch strategy was designed. Through constructing the NanoSwitch, we controlled the switchable CAR‐T cells indirectly without complicated construction of CAR. Considering the sensitivity of immune cells to foreign objects, we chose biocompatible material PEG–poly(*ε*‐caprolactone), which has been applied in US Food and Drug Administration (FDA)‐approved formulations. We screened the sequence PVGLIG from the reported pooled sequencing of peptide library mixtures by cleavage‐site specificity study and successfully inserted the peptide between mPEG and PCL copolymers as a “junction.” Gelatinases are specifically and highly expressed in gastric cancer according to our previous study.^[^ ^11^, ^12^ ^]^ When NPs reached tumor sites, the mPEG–PCL conjugates will be cleaved at the certain site of the peptide, and the dePEGylated NPs will interact with tumor cells more effectively, leading to increased cellular uptake of NPs into tumor cells and tumor‐specific activation of CAR‐T cells.^[^ ^13^ ^]^ There have been several on‐switch strategies to improve efficacy and safety of CAR‐T therapy.^[^ ^22^ ^]^ For example, Stepanov et al.^[^ ^20^ ^]^ designed a barstar‐based CAR. Through Designed Ankyrin Repeat Proteins (DARPin)–barnase proteins targeting HER2, the CAR‐T attacked HER2‐positive cells. However, if normal cells express HER2, they still could not escape the attack of CAR‐T. Actually, the problem of treatment‐related toxicities has not been actually solved, which attribute to the CAR‐T itself.^[^ ^4^ ^]^ Our design is the first attempt to use tumor‐targeting nanocarrier to solve the core hurdles in CAR‐T against solid tumors. More importantly, the NanoSwitch is “universal,” as on one hand, the Switchable CAR‐T can be applied to different tumor types or different targets,on the other hand, the NanoSwitch is not only limited to the gelatinase‐responsive strategies, but also available for other tumor‐targeting drug delivery strategies, and thus of great feasibility and practicality.

CRS is the most common adverse event of CAR‐T therapy. CAR‐T cells engage with targets and activate excessively, resulting in a systemic inflammatory response. Severe and sometimes lethal cytokine storms have been reported in patients treated with CAR‐T cells in many clinical trials,^[^ ^2d^ ^]^ but there have not been effective strategies for the eradication of CRS. Xenogeneic transferred T cells do not usually cause immune cascade reaction, so CRS is not that violent in these preclinical trials. Thus, in the subcutaneous tumor model, only the level of IL‐2 showed significant differences between CAR‐T/NanoSwitch and CAR‐T/FreeSwitch group. Nevertheless, in peritoneal metastasis tumor model, various cytokines especially IL‐6 showed a rise in the CAR‐T/FreeSwitch group. Owing to the sustained‐release effect of switch by NPs, IL‐6 secreted by CAR‐T cells activated by NanoSwitch was significantly steady. Accordingly, our NanoSwitch prevented furious activation of CAR‐T cells, thus has the potential to solve the problem of CRS. We expected that this effect would be more obvious in clinical use if possible. The on‐target/off‐tumor effect is also one of the most prominent hurdles for the application of CAR‐T. We preliminarily confirmed in this study that the NanoSwitch‐activated CAR‐T did not attack nontumoral cells even if they express HER2. However, as HER2 is closely related to a series of physiological activities, the construction of the model was quite difficult. As a result, in the animal model we used in this study, the organs did not express human HER2. We assume that CAR‐T/FreeSwitch will cause more severe side effects, thus the CAR‐T/NanoSwitch will be much safer if used clinically.

In summary, we demonstrate that nanomodified switch activated CAR‐T cells precisely and controllably in vivo, leading to significant improvement in the safety and applicability of CAR‐T therapy. We also look forward to the application of NanoSwitch clinically to test the impact of this feasible strategy on CAR‐T cells in patients with solid tumors.

## Conflict of Interest

The authors declare no conflict of interest.

## Supporting information

Supporting Information

## Data Availability

The data that support the findings of this study are available from the corresponding author upon reasonable request.
