# Corrigendum to “Nanomodified Switch Induced Precise and Moderate Activation of CAR‐T Cells for Solid Tumors”

**DOI:** 10.1002/advs.202501355

**Published:** 2025-02-22

*Adv Sci (Weinh)*. **2023**, 202205044.

https://doi.org/10.1002/advs.202205044

In Figure 4d, histological analyses of liver toxicity through hematoxylin‐eosin (H&E) staining (×200). But the photo at row four and column five was at magnification x100 but not ×200. Please find the picture with the correct magnification below.


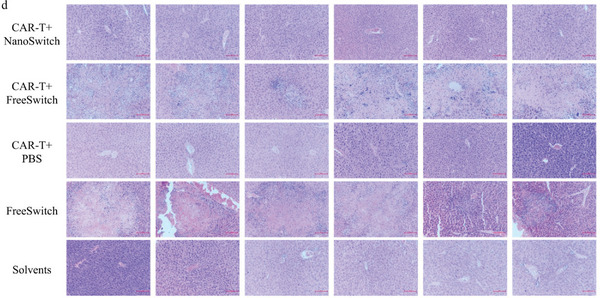


We apologize for this error.

