# Supplementary material for: Nanomodified Switch Induced Precise and Moderate Activation of CAR‐T Cells for Solid Tumors
Source: Adv Sci (Weinh). 2023 Feb 8;10(12):2205044. doi: 10.1002/advs.202205044 (PMC10131841; doi:10.1002/advs.202205044)
Supplement: Supplementary file 1 — Supporting Information [file ADVS-10-2205044-s001.pdf]

## 1. Supplemental materials and methods

### 1.1. Materials and agents

### 1.1 Materials

Polyethyleneglycol-NHS (PEG-NHS, Mn 5000, polydispersity≤1.08) was synthesized by Beijing Jiankai Technology Company (Beijing, China). The gelatinases-cleavable peptide (PVGLIG) was synthesized by Shanghai HD Biosciences Company (Shanghai, China). Poly(ε-caprolactone)-NH2(PCL-NH2, MW:10K)was synthesized by Xi'anruixi Biological Technology Co., Ltd. (Xi'an, China). Cell culture medium and supplements were purchased from Gibco (NY, USA). All other reagents including rapamycin were obtained from Aladdin (Shanghai, China) unless otherwise stated.

### 1.2 Cell lines

Human gastric adenocarcinoma cell line MKN45, human breast adenocarcinoma SK-BR-3, human gastric mucosal epithelial cell line GES-1 were purchased from the Cell Bank of Shanghai Institute of Biochemistry and Cell Biology. Extracellular and transmembrane fragment of human HER2 was added to MKN45 and GES-1 cells by lentiviral gene transfer from ICarTab Biomedical (Suzhou, China), followed by puromycin selection to establish stable cell population of MKN45(HER2+) and GES-1(HER2+). The antigen expression was confirmed by flow cytometry prior to use.

The blood collection procedure was approved by the Ethics Committee of Nanjing Drum Tower Hospital, the Affiliated Hospital of Nanjing University Medical School (Nanjing, China). Peripheral blood mononuclear cells (PBMCs) were isolated from samples from healthy volunteers by density gradient centrifugation on a Ficoll density gradient[1].

### 1.3. Cell culture

The MKN45, SK-BR-3, GES-1 cells were cultivated in RPMI-1640 medium, supplemented with 10% fetal bovine serum and incubated at 37°C in a humidified chamber containing 5% CO2. When the degree of cell fusion reached 80%-90%, the cells were digested with 0.25% trypsin, and then sub-cultured or inoculated in cell plates for subsequent experiments.

The T cells were cultivated in AIM-V medium with 10% fetal bovine serum and incubated at 37°C in a humidified chamber containing 5% CO2. After transfection, the cell culture medium was half replaced by fresh complete medium containing100 U/ml IL-2, 10 ng/ml IL-7 (PeproTech, NJ, USA) and 10 ng/ml IL-15 (PeproTech, NJ, USA) every 2–3 days.

### 1.4. Methods

#### 1.4.1 Preparation and characterization of NanoSwitch

Gelatinase cleavable peptide (PVGLIG) was inserted between mPEG and PCL segment (mPEG-Pep-PCL) as we previously described[2]. Nanoparticles were prepared using a double-emulsion method. Briefly, rapamycin was dissolved in DCM at 10 mg/mL, and then added to PEG-pep-PCL dichloromethane solution. The

mixture was homogenized with 5% w/v polyvinyl alcohol (PVA) solution using probe sonication (XL2000, USA) at 27.5 W for 60 s. This water/oil (w/o) emulsion was transferred to an aqueous solution 1% w/v PVA, and the mixture was probe-sonicated at 17.5 W for 30 s. The resulting w/o/w emulsion was then mechanically stirred for 2 h to remove DCM. The NPs were purified by centrifugation at 15000 rpm for 30 min (Leiboer LG16B centrifuge, China) and reconstituted two times with deionized and distilled water.

Drug-loading content and encapsulation efficiency of the nanoparticles were obtained by the following equations[3]

$$\text{Drug loading content}(\%) = \frac{\text{Weight of the drug in NPs}}{\text{Weight of the drug in NPs} + \text{Weight of the copolymers used}} \times 100\% \quad (1)$$

$$\text{Encapsulation efficiency}(\%) = \frac{\text{Weight of the drug in NPs}}{\text{Weight of the feeding drugs}} \times 100\% \quad (2)$$

**1.4.2 Preparation of switchable CAR-T cells**

The CAR incorporated a single chain variable fragment (anti-HER2 ScFv), a “split” fragment (FKBP, FRB) that can be conditionally re-assembled when a heterodimerizing small molecule agent (rapamycin) is present, a human CD8a hinge and transmembrane domain, an intracellular 4-1BB (CD137) costimulatory domain, a cytoplasmic CD3ζ signal and Enhanced Green Fluorescent Protein (EGFP). (Figure S1a). Sequences encoding the FKBP and the T2089L mutant of FRB domains were obtained from Addgene (plasmids #20160 and #20148). The plasmid was produced by ICarTab Biomedical Co., Ltd (Suzhou, China).

As to the construction of CAR-T, we used PiggyBac transposon system to conduct plasmid transfection as described[4]. After stimulation with 50 ng/ml OKT3 (eBioscience, CA, USA) for 48h, T cells were transfected with the intended plasmids by electroporation (Nucleofector 2B, Lonza, Basel, Switzerland). Briefly, $10^7$ cells were washed with DPBS and resuspended in 100μl transfection buffer (Amaxa Human T cells Nucleofector Kit, VPA-1002, Lonza, Basel, Switzerland). Program T-007 was selected. We monitored the CAR expression level by detecting the expression of Enhanced Green Fluorescent Protein (EGFP) at different time points.

**1.4.3 Preparation of target cells**

We customized the lentivirus from ICarTab Biomedical (Suzhou, China). First, the MKN45 (or GES-1) cells were plated in two 10-cmdishes with complete medium (about 0.5-1$\times10^6$cells/dish). Appropriate amount of lentivirus [at multiplicity of infection of 1 (MOI=1)] were diluted into fresh complete media (contain 6 μg/mL of polybrene) on the next day. The medium in one plate was changed into that containing lentivirus, where cells were incubated overnight. Meanwhile, the other plate which acted as killing control didn't add any lentivirus in the medium. The following day, the

medium containing virus was removed and replaced with fresh and complete culture medium. MKN45 (or GES-1) cells were harvested 72 h after transduction. We establish stable cell population by puromycin selection. Add the puromycin at the killing concentration (2μg/ml) to both plates, and fresh medium with antibiotic is added every two days until all the cells in the killing control plate are dead. Expression of HER2 was quantified through flow cytometry analysis of anti-HER2-PE (Biolegend, CA, USA).

Extracellular and transmembrane fragment sequence of HER2:
MELAALCRWGLLLALLPPGAASTQVCTGTDMKLRLPASPETHLDMLRHLYQ
GCQVVQGNLELTYLPTNASLSFLQDIQEVQGYVLIAHNQVRQVPLQRLRIVRG
TQLFEDNYALAVLDNGDPLNNTTPVTGASPGGLRELQLRSLTEILKGGVLIQR
NPQLCYQDTILWKDIFHKNNQLALTLIDTNRSRACHPCSPMCKGSRCWGESSE
DCQSLTRTVCAGGCARCKGPLPTDCCHEQCAAGCTGPKHSDCLACLHFNHSG
ICELHCPALVTYNTDTFESMPNPEGRYTFGASCVTACPYNYLSTDVGSCTLVCP
LHNQEVTAEDGTQRCEKCSKPCARVCYGLGMEHLREVRAVTSANIQEFAGCK
KIFGSLAFLPESFDGDPASNTAPLQPEQLQVFETLEEITGYLYISAWPDSLPDLS
VFQNLQVIRGRILHNGAYSLTLQGLGISWLGLRSLRELGSGLALIHHNTHLCFV
HTVPWDQLFRNPHQALLHTANRPEDECVGEGLACHQLCARGHCWGPGPTQC
VNCSQFLRGQECVEECRVLQGLPREYVNARHCLPCHPECQPQNGSVTCFGPE
ADQCVACAHYKDPPFCVARCPSGVKPDLSYMPIWKFPDEEGACQPCPINCTHS
CVDLDDKGCPAEQRASPLTSIISAVVGILLVVVLGVVFGILIKRRQQKIRK

### 1.4.4 T cell activation and cytotoxicity assays

CAR-T cells were cocultured with MKN45(HER2+) at an E:T ratio of 10:1. After incubation for 24 h, T cell activation was assessed by flow cytometry analysis of anti-CD69-APC (FN50, Biolegend, CA, USA). The supernatant fluids were harvested for cytokine quantification with IFN-γ cytometric bead array (BD Biosciences, CA, USA) and ELISpot array (Dakewei, Shenzhen, China). For cytotoxicity assays, we firstly stained living target cells with CellTrace™ Far Red Cell Proliferation Kit (C34564, MA, Thermofisher, USA). The red excitation at 630 nm and emission at 661 nm of CellTrace Far Red dye has limited spectral overlap with fluorescent proteins EGFP. The dry CellTrace™ reagents were dissolved in DMSO with stock concentration of 1 mM, which was diluted with pre-warmed (37°C) PBS to working concentration of 1μM before use. We removed the supernatant of MKN45(HER2+) or GES-1(HER2+) cells and resuspended them at $5\times10^6$/ml with working solution. After incubating at room temperature for 20 minutes protected from light, the cells were added five times the original staining volume of culture medium (containing at least 1% protein) and incubated for 5 minutes. The cell populations were washed twice with PBS, resuspended in RPMI-1640 complete medium at a concentration of $2\times10^5$/ml, and spread into a sterile flow tube 200μl per well ($4\times10^4$ per well). We resuspended the CAR-T cells to $2\times10^6$ /mL after washing with PBS, added the effector cells into the sterile flow tube with target cells (E:T ratio = 5:1), and added PBS, NanoSwitch (equivalent to 500 nM) or FreeSwitch (500 nM) respectively in different wells as

comparison, then replenish the RPMI-1640 complete medium to 400μl, and the cells were incubated for 5-6 h. Washing the cells with PBS and resuspending them to 100μl, we added PI (Thermofisher, MA, USA) to the concentration of 0.1μg/mL. Then the cells were incubated in the dark for 10min at room temperature before flow cytometry. The gating methods for flow cytometry analysis of cytotoxicity assays are shown as follows.

**1.4.5 Real-time near-infrared fluorescence imaging**

Bis(trihexylsiloxy)silicon 2,3-naph-thalocyanine (Sigma, MO, USA) was labeled in NPs by double-emulsion method according to the method described in Method 1.4.1. Then unconjugated dye was removed by dialysis (MWCO 3500 Da) for 2 days. Labeled NPs were injected intraperitoneally to Balb/c nude mice. At different time intervals, the mice were anesthetized and scanned using an IVIS Lumina III system (PerkinElmer, Massachusetts, USA).

**1.4.6 In vivo antitumor efficacy**

All animal procedures were carried out in compliance with guidelines set by the Animal Care Committee at Nanjing Drum Tower Hospital, The Affiliated Hospital of Nanjing University Medical School (Nanjing, China). Mice were randomized on the basis of age and weight. 5-week-old female NOD/SCID mice were injected subcutaneously with $1 \times 10^6$ MKN45(HER2+) cells. Treatment was started when tumor volumes reached approximately 100 $mm^3$. Mice were treated with intraperitoneal injection of NanoSwitch (equivalent to FreeSwitch 3mg/kg according to the drug loading content), FreeSwitch (3mg/kg), Blank NP. Then $5 \times 10^6$ CAR-T cells were given via intravenous injection 12h after switch transfer. The above treatment was given every eight days for a total of twice. Tumor size was inspected every other day, calculated by the formula length $\times$ width$^2$ $\times$ 0.5.

**1.4.7 In vivo safety study**

Rapamycin was dissolved in vehicle solution (16.7% 1,2-propanediol, 22.5% PEG-400, 1.25% Tween-80) to 0.6mg/mL prior to injection. The NanoSwitch was diluted to the same concentration according to the drug loading content. The FreeSwitch (3mg/kg) and NanoSwitch (equivalent to FreeSwitch 3mg/kg according to the drug loading content) were given every four days to BALB/c nude mice and the weights were measured every other day. 21 days after treatment, all mice were sacrificed to collect tumor samples and blood serum. The levels of ALT and AST were test in clinical laboratory of Nanjing Drum Tower Hospital, The Affiliated Hospital of Nanjing University Medical School (Nanjing, China). Organs were fixed in 10% neutral-buffered formalin, embedded in paraffin, sliced, and stained with hematoxylin–eosin (H&E).

**1.4.8 In vivo immunological analysis**

For subcutaneous tumor model, mice were injected subcutaneously with $1 \times 10^6$ MKN45(HER2+) cells. Mice were treated with intraperitoneal injection of

NanoSwitch and FreeSwitch (3mg/kg). $1 \times 10^7$ CAR-T cells were given via intravenous injection 12h after switch transfer. Twelve hours later, all mice were sacrificed to collect tumor samples and blood serum. Cytokine was quantified using the Human Th1/Th2 Cytokine Kit (BD Bioscience, CA, USA).

For peritoneal metastasis tumor model, mice were injected intraperitoneally with $5 \times 10^6$ MKN45(HER2+) cells. Mice were treated with intraperitoneal injection of 100 μl PBS, NanoSwitch and FreeSwitch (3mg/kg) as well as $1 \times 10^7$ CAR-T cells. 12 hours later, peritoneal effusion was collected and cytokine was quantified using the Human Th1/Th2 Cytokine Kit (BD Bioscience, CA, USA).

**1.4.9 Multispectral immunofluorescent imaging**

Three days after infusion of CAR-T cells, three mice in every group were sacrificed to collect tumor samples. Fixed in 4% neutral buffered formalin for at least 24 hours, tumor samples were processed for dehydratation and paraffinization. After antigen-repairing, slides were managed for deparaffinization, epitope retrieval and endogenous peroxidase quenching. The multiplex staining method included several rounds of staining[5]. In every round, blocking the non-specific site, slides were incubated with unlabeled primary antibody (CD3: ab231775, CD69: ab233396, Abcam) and then the HRP-conjugated secondary antibody (TalentBio, Shanghai, China). Nuclear staining was performed using Spectral DAPI dye solution (RecordBio, Shanghai, China). Multiplex IF images from the stained slides were acquired through Pannoramic MIDI: 3Dhistech multispectral microscope.

**1.4.10 RNA sequencing and analysis**

Extracted the RNA of transfected CAR-T cells activated by PBS, FreeSwitch (500 nM) and NanoSwitch (equivalent to 500 nM). Extracted the RNA of tumor samples of excised tumor from CAR-T/FreeSwitch and CAR-T/NanoSwitch group in Method 1.4.6 (n=3). Library construction and RNA-sequencing were performed as a fee-for-service by GENEWIZ, Inc. (Suzhou, China). Briefly, after a series of experimental procedures, mRNA libraries were constructed and sequenced on an Illumina HiSeq instrument for sequencing according to manufacturer’s instructions. High-quality sequencing data were obtained with the help of Cutadapt (V1.9.1). DEGSeq(V1.38.0) Bioconductor package(v1.26.0) was used to perform differential expression analysis. The genes with an adjusted *p* value less than 0.05 tested by DEGSeq were considered differentially expressed. Gene Ontology (GO) and Kyoto Encyclopedia of Genes and Genomes (KEGG) enrichment analysis used GOSeq(v1.34.1), and the database (http://en.wikipedia.org/wiki/KEGG) respectively. Protein-protein interaction(PPI) networks were mapped by String (https://cn.string-db.org/, v11.5) and Cytoscape (v3.9.1).

**1.4.11 Statistical Analysis**

The Student’s t-test was used for statistical analyses. P-value style: *p < 0.05; **p < 0.01; ***p <0.001. All statistical analyses were performed using SPSS Statistics 26 software. Graphs were created using GraphPad Prism version 8.00 unless otherwise

stated.

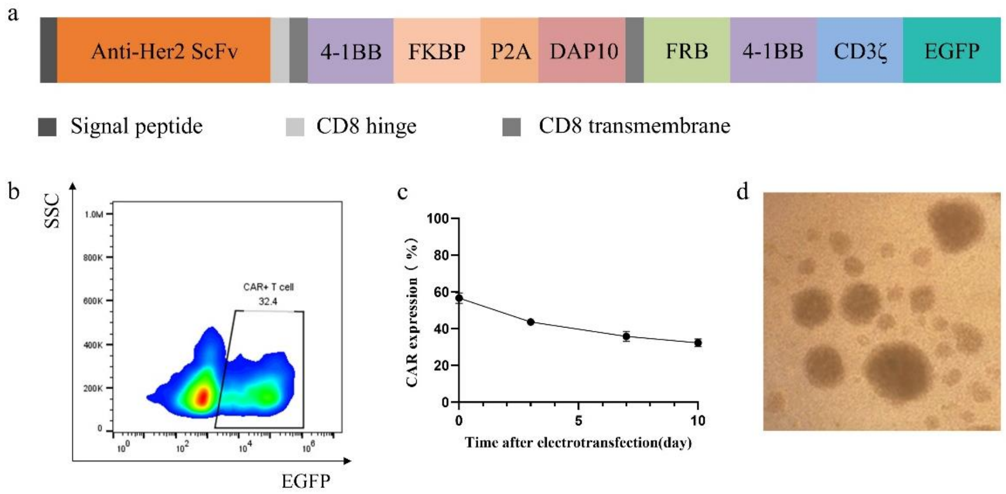


Figure S1. Construction and transfection of switchable CAR-T cells. (a) Schematic illustration of switchable CAR-T. (b) Transfection efficiency of switchable CAR-T cells 7 days after electrotransfection. (c) CAR expressing level was detected the by the EGFP incorporated into the plasmid using flow cytometry at day 0,3,7,10. Data are shown as mean ± SD. Datapoints represent the mean of at least three experiments. Where the error bars are not readily evident, the SD was lower than the width of the symbols. (d) Morphology observation 7 days after electrotransfection indicated that switchable CAR-T cells were in good condition.

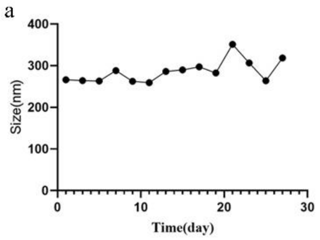

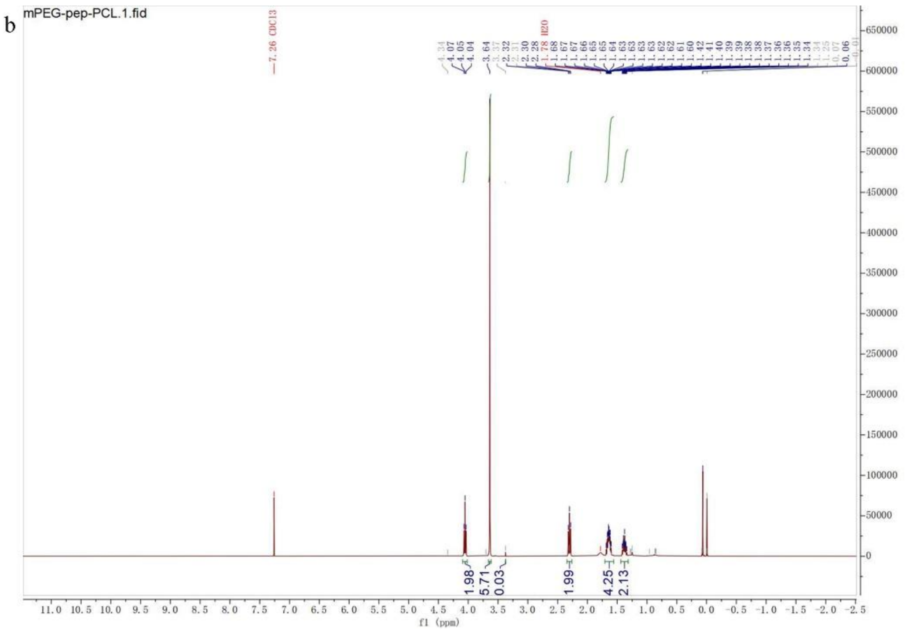


Figure S2. Preparation and characterization of NanoSwitch. (a)Size of NanoSwitch measured by Dynamic light scattering(DLS). (b) $^{1}$H nuclear magnetic resonance spectra (300 MHz, 25°C) of PEG-Pep-PCL in $CDCl_3$. Data are shown as mean ± SD. Datapoints represent the mean of at least three experiments. Where the error bars are not readily evident, the SD was lower than the width of the symbols.

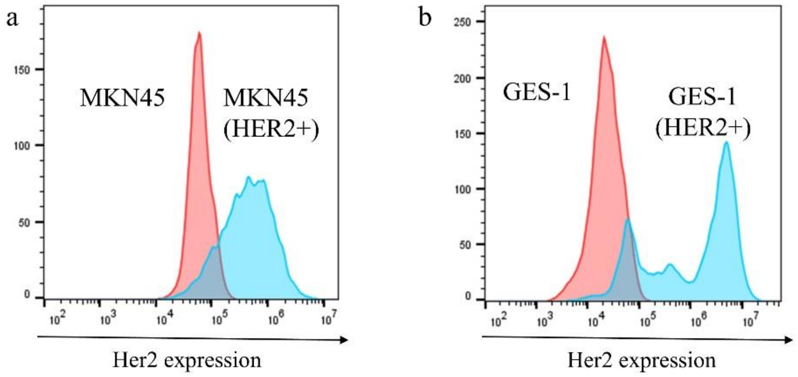


Figure S3. Preparation of target cells with lentivirus system containing overexpressed HER-2 extracellular and transmembrane region. (a) Quantifying expression of Her2 on genetically modified MKN45 cell surface. (b) Quantifying expression of Her2 on GES-1 cell surface.

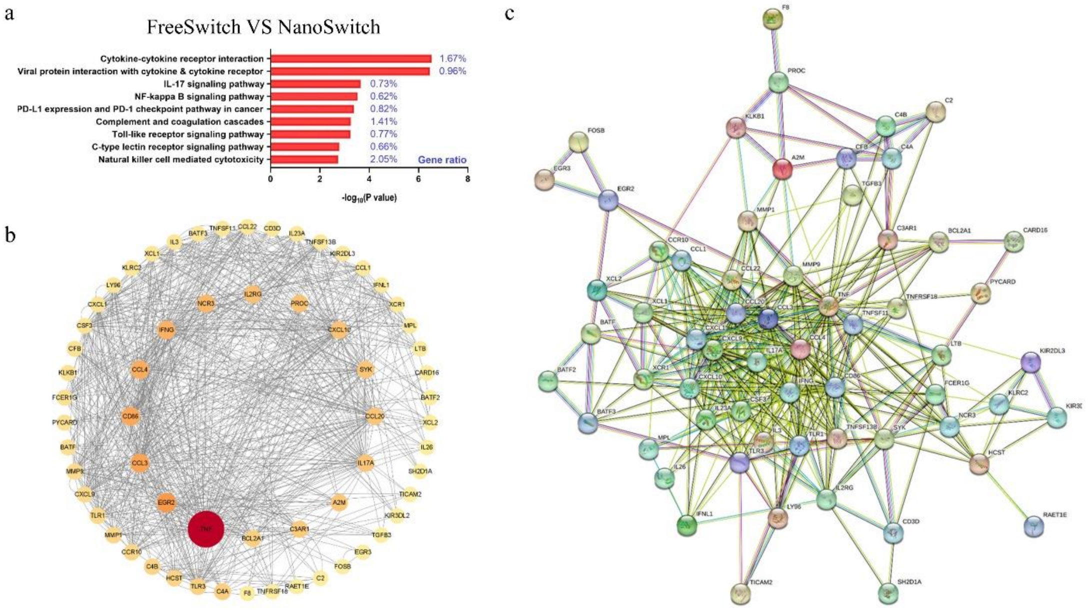


Figure S4. (a) Enriched KEGG signaling pathways relating to tumor immunology. (b-c) Interaction network of genes immune-related genes differentially expressed in the NanoSwitch group compared with the FreeSwitch group analyzed using the Search Tool for the Retrieval of Interacting Genes/Proteins (STRING) algorithm.

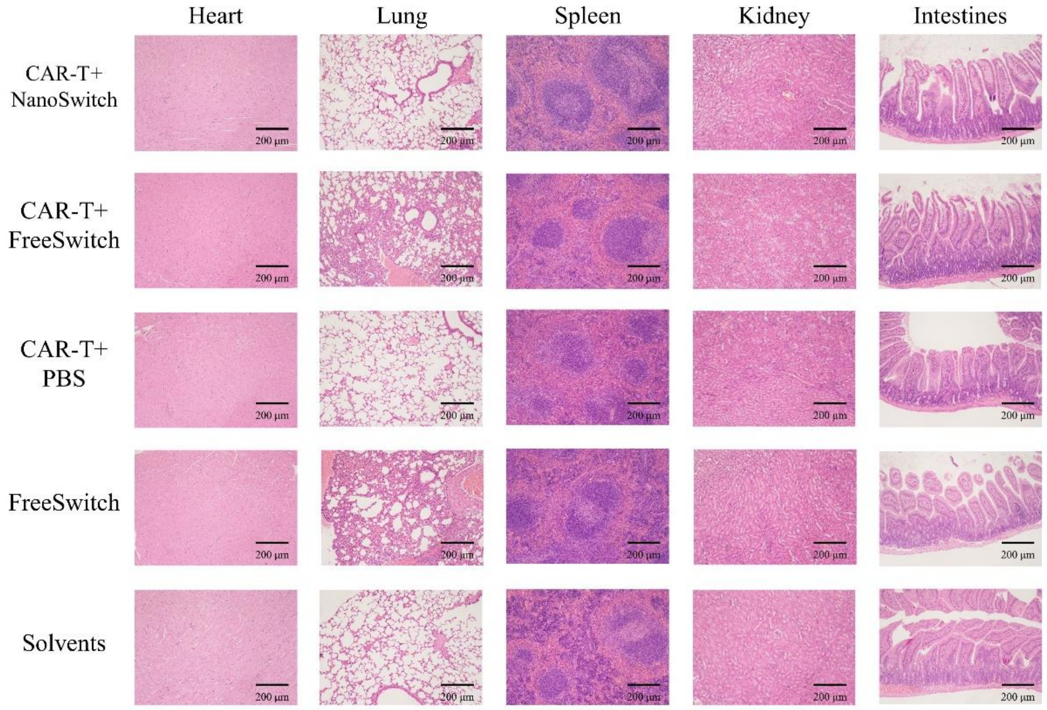


Figure S5. Histological analyses of major organs toxicity through H&E staining (×100) after treatment with CAR-T + NanoSwitch, CAR-T+ Freeswitch, CAR-T+ PBS, Freeswitch, solvents of FreeSwitch respectively. Scale bar: 200 μm.

[1] A. Kizhakeyil, S. T. Ong, M. Fazil, M. L. S. Chalasani, P. Prasannan, N. K. Verma, *Methods Mol Biol* **2019**, 1930, 11.

[2] Q. Wang, Y. T. Yen, C. Xie, F. Liu, Q. Liu, J. Wei, L. Yu, L. Wang, F. Meng, R. Li, B. Liu, *Drug Deliv* **2021**, 28, 510.

[3] R. Li, W. Wu, Q. Liu, P. Wu, L. Xie, Z. Zhu, M. Yang, X. Qian, Y. Ding, L. Yu, X. Jiang, W. Guan, B. Liu, *PLoS One* **2013**, 8, e69643.

[4] Z. Zhang, D. Jiang, H. Yang, Z. He, X. Liu, W. Qin, L. Li, C. Wang, Y. Li, H. Li, H. Xu, H. Jin, Q. Qian, *Cell Death Dis* **2019**, 10, 476.

[5] X. Wang, J. Gao, C. Li, C. Xu, X. Li, F. Meng, Q. Liu, Q. Wang, L. Yu, B. Liu, R. Li, *Mater Today Bio* **2022**, 15, 100305.
